# Metropolitan Poor-Law Infirmaries and Medical Teaching

**Published:** 1887-01-22

**Authors:** 


### Metropolitan Poor-law Infirmaries and Medical Teaching.

By Lieut.-Colonel Montefiore.

**The Old Sick Wards of Workhouses.** Very little more than twenty years have passed since Mr. Farnell and Dr. Smith investigated, on behalf of the Local Government Board, the condition of the sick-wards of the workhouses; and their report was summarised as follows. They found (1) insufficient medical care and treatment; (2) imperfect construction and sanitation; (3) over-crowding; (4) deficient means of classification. For the first point they found, in the large majority of cases, the medical charge of the workhouse was entrusted to an unassisted general practitioner, who was able to devote to the care of the sick such time only as he could spare from his private practice. As for the nursing, the deficiency was even more glaring. It was found that in the metropolitan workhouses 8,360 sick persons were attended during the day by 111 paid nurses. This would give one paid nurse to 75 patients; but this proportion would not represent the shortcoming adequately. No less than 24 of these nurses were employed at St. Marylebone and St. Pancras, leaving 87 paid nurses to attend to 7,840 sick in the other workhouses, which would give one paid nurse to 90 patients. As to paid night-nurses, in the whole of the London workhouses there were three; 38 of the 40 workhouses then occupied relied entirely on pauper aid during the night time. The construction of the sick-wards was nearly all of the corridor type, which allowed of no thorough ventilation. The third point, "over-crowding," was shown to be great, the space allowed per bed being 500 to 600 cubic feet, and in many cases less was given than the smaller figure. On the fourth defect mentioned, the separation between the sick and healthy was found imperfect; cases of infectious diseases were freely admitted into the sick-wards.

**No Progress.** I regret to say I found one Union, Bethnal Green, where the Guardians had not made any great strides towards improvement with regard to the construction of their sick-wards, or in the matter of nursing their sick, the building being of the corridor type, and paupers being employed as assistant nurses. In two other Unions, Hampstead and Lewisham, the sick-wards are contiguous to their workhouses, and they have no resident medical officers, but only one visiting medical man; and the management is under the control of the master and matron of the workhouses.

**Modern Poor-law Infirmaries.** I propose placing the institutions under two heads, for the purpose of giving a very short account of their construction: 1st, those built on the approved pavilion system; 2nd, those irregularly constructed, which have been converted from other buildings into infirmaries, and added to in many cases.

**Pavilion System.** It will be unnecessary for me to give to this audience a definition of the pavilion system. There are 14 institutions built on this plan; they are as follows:—

| | No. of beds certified. | Date of building. |
|---|---|---|
| St. George's, W. ... ... | 776 | ... 1878 |
| St. Marylebone ... ... | 744 | ... 1881 |
| Lambeth ... ... ... | 622 | ... 1878 |
| Holborn ... ... ... | 618 | ... 1879 |
| Poplar & Stepney Sick Asylum | 586 | ... 1871 |
| St. Pancras ... ... ... | 523 | ... 1883 |
| Fulham ... ... ... | 486 | ... 1884 |
| Mile End ... ... ... | 483 | ... 1883 |
| Wandsworth ... ... ... | 447 | ... 1871 |
| St. Olave's ... ... ... | 388 | ... 1879 |
| Chelsea ... ... ... | 386 | ... 1873 |
| Paddington ... ... ... | 284 | ... 1885 |
| Greenwich ... ... ... | 247 | ... 1876 |
| Woolwich ... ... ... | 213 | ... 1872 |
| Total ... ... | 6803 | |

These are for the most part composed of either five or seven blocks of buildings, three or four storeys in height, connected by covered-in but thoroughly ventilated corridors on the ground-floor, and by open iron bridges on the higher storeys. The centre block is employed for administrative purposes, and contains the apartments and offices of the Medical Superintendent, the Assistant Medical Officer, the Matron, and the Steward, on the ground-floor; at the entrance the admission wards for males and females are generally placed. In the basement the kitchen, scullery, and meat stores are found, and in rear of the building is the laundry. The culinary departments and the laundries in these fourteen buildings are certainly very fine. In the first the cooking is by steam, gas, or open fire; in the latter the washing is carried on by steam processes, every modern invention being employed. In the blocks on each side of the centre one are the wards, those for males on one side, and those for females on the other. The wards, when single, will hold 28 to 32 beds; when double, 56 to 64 beds. The space allotted to each bed is 850 cubic feet. On each floor or flat of a block there are ordinarily two large single wards, of 28 or 32 beds each; two small separation wards, capable of holding two or four beds each; two head-nurses' sitting-rooms, each with a window looking into the big ward; two ward kitchens, which are chiefly used for warming food, boiling water, etc., but not for cooking; a clean linen store; two lifts, one for people, the other for food; also a patients' day-room. At the outer end of the large wards, built out at the angles, are the bath-rooms and lavatories on one side, the water-closets on the other. There are several exceptions to the rule, as at St. George's, Hanover Square, Holborn, Paddington, and Greenwich, where there are no day-rooms for the patients, and at Lambeth, St. Pancras, and Wandsworth, where there are day-rooms, but they are partitioned off from the wards by a lath and plaster wall, which does not reach the ceiling; this, in my opinion, is not a good plan, (1) as the air of the day-room and the ward is necessarily the same; (2) as those who are sufficiently convalescent to make use of the day-rooms are apt, by their conversation, to disturb the sick in the wards. The Lambeth, Holborn, and Mile-End Infirmaries, and the Poplar and Stepney Sick Asylum, have double wards; and I have heard it remarked that these very large wards cannot be thoroughly ventilated, as their width is so great as to prevent a free current of air passing through their centres. The decision of this point I leave to experts.

**Irregularly built Institutions.** I will now pass on to those Infirmaries under my second head. Of these there are eleven:—

| | Certified No. of beds. | Date of building. |
|---|---|---|
| St. Saviour's* ... | 1,010 | 1850, 1871, 1877 |
| Whitechapel ... ... | 689 | 1876, 1877 |
| City of London ... | 645 | 1849 |
| Kensington ... ... | 592 | 1871, 1880 |
| Islington ... ... | 543 | 1871 |
| Shoreditch ... ... | 472 | 1850, 1872 |
| Hackney ... ... | 437 | 1846, 1880 |
| St. George's, East ... | 332 | 1871 |
| Central London Sick Asylum | 264 | 1850, 1874 |
| Camberwell ... ... | 232 | 1871 |
| Hampstead ... ... | 176 | New part, 1884 |

We have in these irregularly built infirmaries 5,392 beds, making a total, for the twenty-five institutions we have enumerated, of 12,195 beds.

* The St. Saviour's Guardians have built at Champion Hill, Dulwich, a magnificent infirmary, with every modern improvement, for 726 patients, at an estimated cost of about £120,000, which they hope to open this year.

**The Nursing of the Patients.**

I now come to a very important part of the subject—the nursing of the inmates of these Poor-law Infirmaries. It will be found to have improved enormously on the old condition of things; but yet I think it leaves work for further consideration. I found that, out of twenty-five institutions, at twelve the matrons were trained as nurses; at thirteen they were untrained; at four some of the nurses were trained; at thirteen the nurses had been for one year at some other asylum or institution; at eight the nurses were nearly all quite untrained. The average proportion of nurses to patients may be taken roughly as one nurse to twenty to twenty-one patients. The nurses' accommodation I found to be as follows: At ten institutions, good; nearly all the nurses have separate dormitories, and there are mess and recreation rooms. At eight institutions, moderate; nurses sleep three or four or more in one room; sitting, recreation, and mess room all in one. At seven institutions, bad; head-nurses' sitting-room and bedroom in one, attached to ward, and this is in some cases also used as the ward kitchen; no mess or recreation rooms. The laundry work and scrubbing: at eight infirmaries the laundry is worked by paupers; at six the scrubbing is done by paupers.

**Expenditure.**

I have taken the expenditure of the Poor-law Infirmaries from the Local Government Board reports for 1883-4; and in that report only twenty-one of these institutions are given; the following being omitted, Paddington and Fulham, because they were non-existent at that time; Islington, Hampstead, and Lewisham, because the accounts are rendered with those of their workhouses. The total average number of inmates maintained daily during the year was 9.412.9, and the total expenditure during the year was £319,322. This would make the total expenditure for the 12,195 beds about £415,000. The average cost per head per annum is given as £35 12*s*. 5*d*. The cheapest, St. Saviour's, is £20 11*s*. 8*d*. per head per annum. The most expensive, Central London Sick Asylum, is £48 3*s*. 6*d*. per head per annum.

**Prices paid for Food.**

The extraordinary difference in the prices paid by the Guardians in the various Unions may be shown by quoting the highest and lowest prices of a few articles. Let us take bread, candles, eggs, bacon, cheese, and potatoes. Bread, highest, 14*s*. per cwt.; lowest, 9*s*. 4*d*. Bacon, highest, £5 13*s*. 4*d*. per cwt.; lowest, £3 1*s*. 6*d*. Candles, highest, 9*s*. 6*d*. per 12 lbs.; lowest, 1*s*. 6*d*. Cheese, highest, £3 19*s*. 4*d*. per cwt.; lowest, £2 13*s*. Eggs, highest, 10*s*. 6*d*. per 120; lowest, 8*s*. 4*d*. Potatoes, highest, £6 per ton; lowest, £3 15*s*. Other remarkable differences could be shown, but time will not permit me to quote more.

**Medical Officers.**

Each Infirmary has two medical officers; one, the medical superintendent, the other, is his assistant. The latter has in fifteen institutions to dispense drugs, to receive and issue all drugs and surgical appliances; in eleven of these without any assistance whatsoever; in the four others, with occasional assistance; in ten institutions there are paid dispensers. In many instances, the officer has to visit the sick in the adjacent workhouse, as well as to assist his superintendent in the infirmary.

**The Medical Superintendent.**

I should like to say a good deal about the medical superintendents of these infirmaries, but must content myself with only a few remarks. I must especially point out the enormous amount of work they have to perform, and the great responsibility they have to bear. In some cases they have 500 patients to attend to daily; 50 or more of these may, perhaps, be suffering from acute illness. They have to admit new cases, to discharge those able to go out, to give lectures to the nurses, to see that the patients' diet-sheets are correctly kept, to keep up the medical books and registers, also to make *post mortem* examinations when they are necessary, and numberless other things connected with the medical branch of their duties. But, again, these officers have to supervise every detail of business that goes on in these infirmaries, and so they were altogether over-worked at present.

**The Infirmaries and Medical Education.**

Now we come to the importance of throwing open these institutions in such a manner as to enable medical science to benefit by the better investigation of the diseases of interest that may be found amongst these 12,000 patients. I found, when I visited these institutions last year, that very many of the medical superintendents had put schemes before their boards of guardians, by which some arrangements might be arrived at to allow of more copious notes being taken of cases, and in some instances to admit students to the infirmaries.

**Changes recently Introduced.**

It may be well if I relate, as briefly as I can, what has taken place in some Unions with regard to this matter. The Assistant Medical Officer of the St. Pancras Infirmary resigned, whereupon the Guardians proposed, and the Local Government Board have sanctioned, that two Resident Medical Officers be appointed to take his place, those gentlemen to be chosen from those who have recently passed with distinction through the school of the University College Hospital. They are to hold the appointment for one year, and to receive an honorarium of £50 each, with free board and lodging. It must be noted that the University College Hospital is the largest general hospital in the St. Pancras Union. The St. Marylebone Guardians have requested the Local Government Board to allow them to appoint a Resident Clinical Assistant, in addition to their present medical staff. At the Whitechapel Infirmary the Medical Superintendent resigned, and a proposal was placed before the Guardians, which, however, they did not sanction, that the post should be refilled, but that the London Hospital should provide a non-resident Consulting Physician and Surgeon, who should be responsible for the treatment of the sick, the Resident Medical Officers only carrying out their instructions. The document explanatory of this proposal, written by Mr. Vallance, the able clerk of this Union, is extremely interesting, but time will not permit me to give it in full, and as quotation would only be mutilation, I must perforce leave it. The Guardians of the parish of Paddington have, I believe, asked the Local Government Board to allow them to appoint a Resident Clinical Assistant, but I regret to say the appointment is only to take place at the expense of two head-nurses. This, I think, is a mistake, as none of these institutions is over-nursed.

**Attitude of the Medical Superintendents.**

At a meeting of the Society of Poor-law Infirmary Medical Superintendents a resolution was passed to the following effect:—"That each Medical Superintendent should endeavour to induce his Board of Guardians to obtain the sanction of the Local Government Board to the appointment of one or more Resident Clinical Assistants, and also to allow the Medical Superintendent to call in a consultant whenever he thinks it necessary to do so."

**A Course Suggested.**

Now, I cannot but think that the best course to be pursued is, that every large general hospital should link to itself the Poor-law infirmary in its own district, and that the hospital should provide the Guardians with a list of names of those who have lately passed with honour through the school, from which they may select officers for any vacant medical post; also, that the hospitals should authorise any of their full staff to act as consultants to the infirmary, such consultants to receive a fee. The medical superintendent should inform the staff of the hospital of any cases of more than usual interest that there may be in the infirmary, and arrange for any one person on the senior staff bringing with him a class of students to investigate those cases. That the Guardians be induced to give every assistance to their medical staffs, to allow of the due record of cases being taken. I feel very sure, to arrive at any fulfilment of some scheme of this description, it will take much time, and a large amount of caution and patience on the part of all who wish to see it brought to a favourable issue; for any combined plan sent up by the Guardians to the Local Government Board would frighten that Board, and they would not sanction it.

The average Guardian's View

Again, the average Guardian says, "We will not have medical teaching in our infirmaries; the inmates dislike it, and they are not like hospital patients, who are not forced to enter, but do so voluntarily; but our sick are forced to come to us by the inexorable force of destitution." He also says, "We care nothing for the improvement of medical science." I am, therefore, of opinion that it is the duty of every individual medical superintendent to do his utmost to arouse among his Guardians a wish to improve the medical treatment of the sick, and to add to the present staff of the infirmary for that purpose. This would allow of notes being made of cases which have hitherto been left unrecorded, on account of the paucity of the medical officers, and the enormous amount of work that it has been their task to perform. Any help that can be given in this direction by well-known Societies, such as the College of Physicians, the College of Surgeons, or the Hospitals Association, would do much to hasten the desired end.

